# Optimization of Gate-Head-Top/Bottom Lengths of AlGaN/GaN High-Electron-Mobility Transistors with a Gate-Recessed Structure for High-Power Operations: A Simulation Study

**DOI:** 10.3390/mi15010057

**Published:** 2023-12-27

**Authors:** Woo-Seok Kang, Jun-Hyeok Choi, Dohyung Kim, Ji-Hun Kim, Jun-Ho Lee, Byoung-Gue Min, Dong Min Kang, Jung Han Choi, Hyun-Seok Kim

**Affiliations:** 1 Division of Electronics and Electrical Engineering, Dongguk University-Seoul, Seoul 04620, Republic of Korea; kws1117@dongguk.edu (W.-S.K.); junhyeok6293@dgu.ac.kr (J.-H.C.); ehgudakr@dongguk.edu (D.K.); kjsuk0105@dongguk.edu (J.-H.K.); steve1211@dgu.ac.kr (J.-H.L.); 2 Electronics and Telecommunications Research Institute, Daejeon 34129, Republic of Korea; minbg@etri.re.kr (B.-G.M.); kdm1597@etri.re.kr (D.M.K.); 3 Photonic Components Department, Fraunhofer Heinrich-Hertz Institute, Einsteinufer 37, 10587 Berlin, Germany; jung-han.choi@hhi.fraunhofer.de

**Keywords:** gallium nitride, high-electron-mobility transistor, gate-head, gate-recessed, breakdown voltage

## Abstract

In this study, we propose an optimized AlGaN/GaN high-electron-mobility transistor (HEMT) with a considerably improved breakdown voltage. First, we matched the simulated data obtained from a basic T-gate HEMT with the measured data obtained from the fabricated device to ensure the reliability of the simulation. Thereafter, to improve the breakdown voltage, we suggested applying a gate-head extended structure. The gate-head-top and gate-head-bottom lengths of the basic T-gate HEMT were symmetrically extended by 0.2 μm steps up to 1.0 μm. The breakdown voltage of the 1.0 μm extended structure was 52% higher than that of the basic T-gate HEMT. However, the cutoff frequency ($f_{T}$) and maximum frequency ($f_{max}$) degraded. To minimize the degradation of $f_{T}$ and $f_{max}$, we additionally introduced a gate-recessed structure to the 1.0 μm gate-head extended HEMT. The thickness of the 25 nm AlGaN barrier layer was thinned down to 13 nm in 3 nm steps, and the highest $f_{T}$ and $f_{max}$ were obtained at a 6 nm recessed structure. The $f_{T}$ and $f_{max}$ of the gate-recessed structure improved by 9% and 28%, respectively, with respect to those of the non-gate-recessed structure, and further improvement of the breakdown voltage by 35% was observed. Consequently, considering the trade-off relationship between the DC and RF characteristics, the 1.0 μm gate-head extended HEMT with the 6 nm gate-recessed structure was found to be the optimized AlGaN/GaN HEMT for high-power operations.

## 1. Introduction

GaN-based high-electron-mobility transistors (HEMTs), leveraging their superior material properties, such as a wide energy bandgap (3.4 eV) and a high critical electric field (~3.3 MV/cm), have been studied for applications demanding high-power and high-frequency capabilities [1,2,3]. Moreover, a channel layer with a two-dimensional electron gas (2-DEG) is formed in the AlGaN/GaN heterojunction owing to spontaneous and piezoelectric polarization effects [4], which improve the output current density and power amplification characteristics of the device. The AlGaN/GaN HEMTs are widely used in power electronics applications, including high-power and high-frequency operations, owing to these characteristics [5,6,7]. Various field plate (FP) structures in GaN-based HEMTs have been mainly used under high-power conditions. The FP structures redistribute concentrated electric fields at the drain-side gate edge when high voltage is applied, thereby improving the breakdown voltage ($V_{BD}$) [8,9]. Additionally, we symmetrically increased the gate-head-top ($L_{Gate\text{-}Head\text{-}Top}$) and gate-head-bottom ($L_{Gate\text{-}Head\text{-}Bottom}$) lengths of the AlGaN/GaN HEMT in this study. Subsequently, we confirmed that $V_{BD}$ improved considerably without the gate-foot length ($L_{Gate\text{-}Foot}$) of 0.18 μm and the other epitaxial layers changing. Additionally, increases in $L_{Gate\text{-}Head\text{-}Top}$ and $L_{Gate\text{-}Head\text{-}Bottom}$ inevitably generated additional parasitic capacitances, which degrade the cut-off frequency ($f_{T}$) and maximum frequency ($f_{max}$) characteristics [10,11,12]. Employing the gate-recessed structure can prevent the degradation of frequency characteristics, as it increases transconductance ($g_{m}$) and reduces effective barrier height [13,14,15].

To derive the optimized gate structure, we simulated the DC and RF characteristics by extending $L_{Gate\text{-}Head\text{-}Top}$ and $L_{Gate\text{-}Head\text{-}Bottom}$, and increased the gate-recessed depth of various structures. First, the simulated drain current-gate voltage ($I_{DS}$-$V_{GS}$) transfer and frequency ($f_{T}$ and $f_{max}$) characteristics were matched with the corresponding measured data obtained from a 0.18 μm T-gate AlGaN/GaN HEMTs to ensure the reliability of the results. Subsequently, $L_{Gate\text{-}Head\text{-}Top}$ and $L_{Gate\text{-}Head\text{-}Bottom}$ were symmetrically increased up to 1.0 μm, thereby improving the DC characteristics, especially $V_{BD}$. However, frequency characteristics were inevitably degraded. Therefore, we employed an additional gate-recessed structure to minimize the degradation of $f_{T}$ and $f_{max}$. The gate-recessed structure was applied to the 1.0 μm gate-head extended HEMT, which achieved the largest $V_{BD}$, and the thickness of the 25 nm AlGaN barrier layer was thinned down to 13 nm by 3 nm steps to form the gate-recessed structure. Consequently, we determined the optimized gate electrode structure that achieves high breakdown voltage and minimizes the degradation of frequency characteristics.

## 2. Materials and Methods

**Figure 1:**
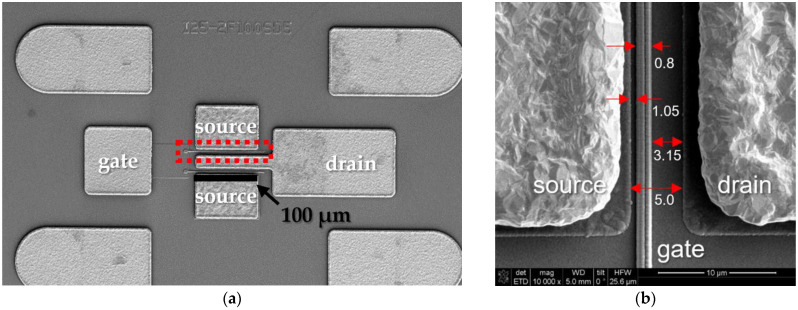
Scanning electron microscope image of the fabricated 0.18 μm T-gate AlGaN/GaN high-electron-mobility transistor structure: (**a**) A top view of the two-finger transistor device. The red dotted frame shows the unit device. (**b**) A top view of the unit device with specific dimensions.

The top view of the fabricated two-finger transistor of the 0.18 μm T-gate AlGaN/GaN HEMT is displayed in Figure 1a. The contact pads at the left and right centers of the figure act as the gate and drain electrodes, respectively, and two source electrodes are located above and below the drain electrodes.

The red dotted frame in Figure 1a shows the scanning electron microscope image of the unit device consisting of the gate, source, and drain electrodes, and the width of the unit device is 100 μm. Figure 1b depicts the specific dimensions of the unit device of the 0.18 μm T-gate AlGaN/GaN HEMT. The source-to-drain ($L_{Source\text{-}Drain}$), gate-to-source ($L_{Gate\text{-}Source}$), and gate-to-drain ($L_{Gate\text{-}Drain}$) lengths of the unit device are 5, 1.05, and 3.15 μm, respectively.

**Figure 2:**
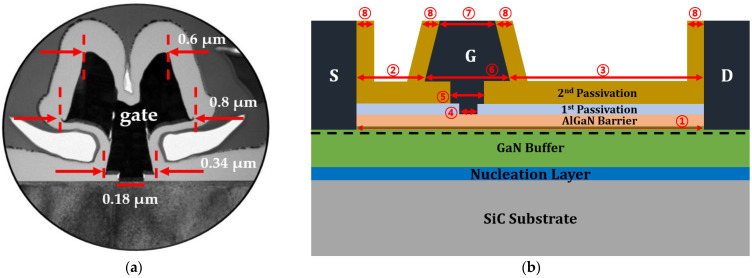
0.18 μm T-gate AlGaN/GaN high-electron-mobility transistor structure: (**a**) a cross-sectional transmission electron microscope image of the gate electrode and (**b**) a cross-sectional schematic illustration of the unit device structure used for modelling.

Figure 2a displays a cross-sectional transmission electron microscope image corresponding to the portion in the red dotted frame in Figure 1a. The T-shaped gate electrode consists of three parts, i.e., $L_{Gate\text{-}Foot}$, gate-middle length ($L_{Gate\text{-}Middle}$), $L_{Gate\text{-}Head\text{-}Top}$, and $L_{Gate\text{-}Head\text{-}Bottom}$, which are 0.18, 0.34, 0.6, and 0.8 μm, respectively. A cross-sectional schematic of the basic T-gate structure used in the model is shown in Figure 2b, where S, G, and D stand for the source, gate, and drain. The detailed geometrical parameters of the structure are listed in Table 1.

The AlGaN/GaN heterostructure HEMT was grown on a 4-inch SiC substrate using metal-organic chemical vapor deposition. The epitaxial layers were stacked in the growth sequence in the following order: a nucleation layer with a thickness of 0.2 µm, an Fe-doped GaN buffer layer with a thickness of 2 µm, and an AlGaN barrier layer with a thickness of 25 nm with a nominal Al composition of 25.5%. The Ti/Al/Ni/Au alloyed ohmic contacts required for the source and drain were formed by rapid thermal annealing at 900 °C for 30 s, and device isolation involved P+-ion implantation. Subsequently, a 50 nm SiN layer was deposited on the AlGaN barrier using plasma-enhanced chemical vapor deposition (PECVD). The alloyed ohmic source and drain contacts were interconnected via evaporated Ti/Au metals after etching the SiN layer. The 0.18-μm-width $L_{Gate\text{-}Foot}$ was first defined via electron beam exposure to a polymethyl methacrylate (PMMA) resist, and the SiN layer beneath the gate pattern was formed via reactive ion etching (RIE). Subsequently, a 0.34 μm wide $L_{Gate\text{-}Middle}$ was formed using additional electron beam exposure after a triple-layer-coating of PMMA/co-polymer/PMMA. The two-step gate-recess to define $L_{Gate\text{-}Foot}$ and $L_{Gate\text{-}Middle}$ was formed via dry etching using an inductively coupled plasma with a BCl_3_/Cl_2_ gas mixture and a wet cleaning process using oxygen plasma treatment, followed by diluted-HCl etching. To form the T-shaped gate electrode, an Ni/Au metal stack with Au and Ni thicknesses of 30 and 500 nm, respectively, was deposited via electron beam evaporation and lifted off. Finally, SiN was deposited via PECVD for device passivation and subsequently etched using RIE to form the ohmic electrode-pad contacts. Further information regarding the fabrication process of the AlGaN/GaN heterostructure HEMT is available in a previous study [16].

Acceptor trap doping was applied in the GaN buffer layer using Fe to improve V_BD_ by minimizing the substrate leakage current and preventing electron-induced punch through [17,18,19]. The peak concentration of acceptor trap doping was $10^{18}/{cm}^{3}$, which decreased gradually following a Gaussian doping profile [20]. The acceptor doping concentration in the AlGaN/GaN interface region was set to $6.376\times 10^{16}/{cm}^{3}$ based on values reported in a previous study [21].

The conduction band energy level of a basic T-gate structure was simulated as a function of depth. When AlGaN and GaN come in contact, the top and bottom of the AlGaN layer is negatively and positively charged, respectively, due to the polarization effect occurring in the AlGaN/GaN interfaces. Owing to polarization charges at the top and bottom of the AlGaN layer, an electric field is generated, and energy band bending is induced toward the AlGaN/GaN interface. The electric field causes the electrons in the conduction band of the AlGaN layer to move toward the positively charged AlGaN/GaN interface and accumulate. Subsequently, the accumulated electrons in the AlGaN layer flow into the GaN layer and become confined until the Fermi levels of AlGaN and GaN become equal, thereby forming the 2-DEG. Therefore, the 2-DEG with a density of $5.67\times 10^{12}/{cm}^{2}$ is formed in the heterojunction interface between AlGaN and GaN [21].

To ensure the reliability of the simulation, appropriate parameters and models must be applied for each material. Because the heat generated during device operation degrades the device performance, the self-heating effect (SHE) must be considered. The equation of the thermal conductivity model can be expressed as follows:

(1) $$k\left(T_{L}\right)=(TC.CONST)/{\left(\frac{T_{L}}{300}\right)}^{TC.NPOW},$$

where $T_{L}$ is the local lattice temperature, TC.CONST is the thermal conductivity constants of the material at 300 K, and TC.NPOW is the thermal conductivity factor, which represents the temperature-dependent thermal conductivity, respectively [22,23]. Based on the calculated thermal conductivity model, the lattice heat flow model is given by:

(2) $$C\frac{\partial T_{L}}{\partial t}=\nabla \left(k\nabla T_{L}\right)+H,$$

where C, $T_{L}$, and k are the heat capacitance per unit volume, local lattice temperature, and thermal conductivity, respectively [24,25,26], and H is the generated heat, which can be expressed as follows:

(3) $$H=(\vec{J_{n}}+\vec{J_{p}})\cdot \vec{E},$$

where $\vec{J_{n}}$, $\vec{J_{p}}$, and $\vec{E}$ are the electron current density, hole current density, and electric field, respectively [27]. By considering Equations (1)–(3), the SHE can be applied to the simulation by setting proper values for each parameter [28]. In addition, GANSAT and FMCT electron-mobility models, Shockley–Read–Hall recombination, Auger recombination, Selberherr’s models, and the Fermi–Dirac distribution function were considered in the simulation for all layers [29,30]. The values of specific parameters applied to the simulation are listed in Table 2 [31,32].

We analyzed the frequency characteristics to prevent the deterioration of RF characteristics when the gate-head structure is changed. Current gain and unilateral power gain were used to calculate $f_{T}$ and $f_{max}$, respectively. $f_{T}$ can be expressed as follows:

(4) $$f_{T}=\frac{g_{m}}{2\pi (C_{gs}+C_{gd})}\approx \frac{g_{m}}{2\pi C_{gs}},$$

where $g_{m}$ is the transconductance, and $C_{gs}$ and $C_{gd}$ are the gate-to-source and gate-to-drain capacitances, respectively. Parasitic capacitances such as $C_{gs}$ and $C_{gd}$ have an inverse relationship with f_T_, as per Equation (4). Therefore, $C_{gs}$ and $C_{gd}$ must be decreased to increase $f_{T}$. We can also determine $f_{max}$ by applying the calculated value of $f_{T}$, and $f_{max}$ can be expressed as:

(5) $$f_{max}=\frac{f_{T}}{2\sqrt{\pi f_{T}C_{gd}\left(R_{s}+R_{g}+R_{gs}+2\pi L_{s}\right)+G_{ds}(R_{s}+R_{g}+R_{gs}+\pi f_{T}L_{s})}}\approx \sqrt{\frac{f_{T}}{8\pi R_{g}C_{gd}}},$$

where $R_{s}$, $R_{g}$, and $R_{gs}$ are the source, gate, and gate-to-source resistances, respectively; $L_{s}$ is the source inductance; and $G_{ds}$ is the output conductance. Equations (4) and (5) can be used to confirm that $C_{gs}$ and $C_{gd}$ are crucial for determining the values of $f_{T}$ and $f_{max}$, and capacitance can be expressed as:

(6) $$C=\frac{\varepsilon A}{d},$$

where ε, A, and d are the permittivity of the dielectric constant, the area of the plate overlaps in square meters, and the distance between plates in meters, respectively. The distance between the electrodes can be increased, the area of the electrode can be reduced, or a low dielectric constant material used for passivation can reduce capacitance.

## 3. Results

### 3.1. Matching the Measured and Simulated Data Obtained from the Basic T-Gate HEMT

**Figure 3:**
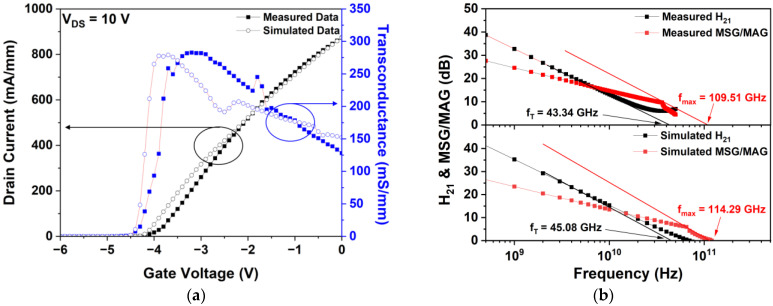
Comparison between the measured and simulated results: (**a**) drain current-gate voltage ($I_{DS}$-$V_{GS}$) transfer characteristics, (**b**) cut-off frequency, and maximum frequency of a basic T-gate HEMT at a drain voltage ($V_{DS}$) = 10 V and a gate voltage ($V_{GS}$) = −3 V.

In this study, we first matched the measured and simulated data obtained from an actual fabricated basic T-gate HEMT to ensure the reliability of the simulation. The simulated drain current–gate voltage ($I_{DS}\text{–}V_{GS}$) transfer characteristics, $f_{T}$, and $f_{max}$ were matched with the corresponding measured values. Figure 3a shows the overlapping values of the measured and simulated $I_{DS}\text{–}V_{GS}$ transfer characteristics at a drain voltage ($V_{DS}$) of 10 V and gate voltage ($V_{GS}$) in the range of −6–0 V. The DC characteristics of the actual fabricated device were measured using an HP4142B modular DC source/monitor probe station (Keysight, Santa Rosa, CA, USA) and a Summit 12000 probe station (Cascade Microtech, Beaverton, OR, USA). The measured and simulated threshold voltages ($V_{th}$) were equal, i.e., −4.3 V, and the measured and simulated maximum transconductances ($G_{m}$) were 282.75 and 278.97 mS/mm, respectively, which were well-matched with a 1.35% error rate. At a $V_{GS}$ of 0 V, the measured and simulated drain currents ($I_{dss}$) were 873.40 and 873.46 mA/mm, respectively, which were also well-matched with only an error rate of 0.006%.

Figure 3b shows the measured and simulated $f_{T}$ and $f_{max}$ at $V_{GS}$ of −3 V and $V_{DS}$ of 10 V. The small-signal RF performance of the fabricated basic T-gate HEMT was measured using a PNA-X N5245A network analyzer (Keysight, Santa Rosa, CA, USA) in the frequency range of 0.5–50 GHz. Furthermore, $f_{T}$ was extracted by extrapolating the current gain ($H_{21}$) to 0 dB using a −20 dB/decade slope, and f_max_ was obtained from the extrapolation of the maximum stable gain/maximum available gain to unity using the same slope [33]. The measured and simulated $f_{T}$ of the basic T-gate HEMT were 43.34 GHz and 45.08 GHz, and $f_{max}$ were 109.51 GHz and 114.29 GHz, respectively. The measured and simulated values of $f_{T}$ and $f_{max}$ were confirmed to be well-matched with the error rates of 4.01% and 4.36%, respectively.

### 3.2. Comparative Analysis of Basic T-Gate and Gate-Head Extended HEMTs

**Figure 4:**
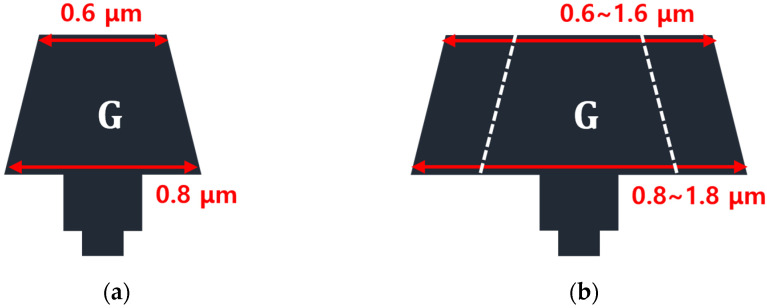
Schematics of the gate electrode structures of the 0.18 μm T-gate AlGaN/GaN HEMT: (**a**) basic T-gate HEMT and (**b**) gate-head extended HEMT, where the white dashed lines represent the initial size of gate-head before extension.

To improve the $V_{BD}$ characteristics, we varied the gate-head extended length. We increased $L_{Gate\text{-}Head\text{-}Top}$ and $L_{Gate\text{-}Head\text{-}Bottom}$ of the basic T-gate HEMT, which were originally 0.6 and 0.8 μm, respectively. Additionally, $L_{Gate\text{-}Head\text{-}Top}$ and $L_{Gate\text{-}Head\text{-}Bottom}$ were symmetrically extended in 0.2 μm steps up to 1.0 μm, as shown in Figure 4.

**Figure 5:**
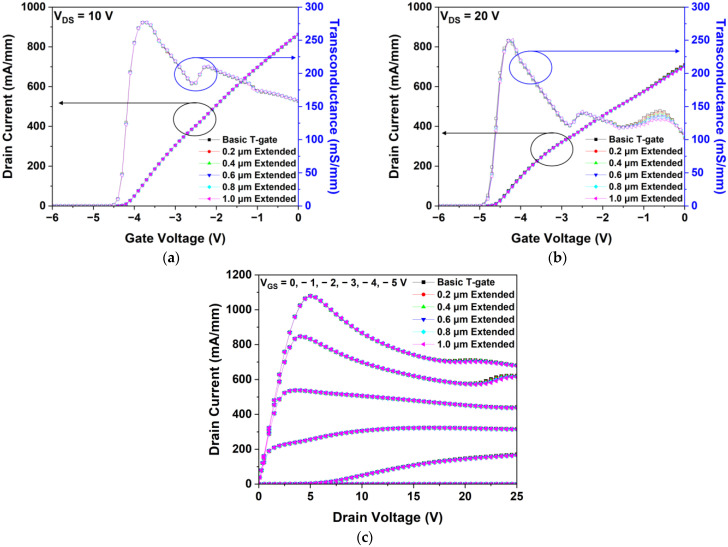
Simulation of the DC characteristics of the gate-head length extended structures: $I_{DS}$-$V_{GS}$ transfer characteristics at (**a**) $V_{DS}$ = 10 V and (**b**) $V_{DS}$ = 20 V and (**c**) drain current-drain voltage ($I_{DS}$-$V_{DS}$) output characteristics at gate voltages of −5, −4, −3, −2, −1, and 0 V.

A gate-head extended HEMT was modeled by increasing $L_{Gate\text{-}Head\text{-}Top}$ and $L_{Gate\text{-}Head\text{-}Bottom}$ symmetrically until the sidewall passivation of the source electrode and the gate electrode contacted. Except for $L_{Gate\text{-}Head\text{-}Top}$ and $L_{Gate\text{-}Head\text{-}Bottom}$, all the remaining geometrical parameters of the device were maintained. The initial values of $L_{Gate\text{-}Foot}$ and $L_{Gate\text{-}Middle}$ were 0.18 and 0.34 μm, respectively. The structures were named as 0.2 μm extended, 0.4 μm extended, 0.6 μm extended, 0.8 μm extended, and 1.0 μm extended HEMTs, according to the extended lengths of the gate-head. Figure 5 explains the schematics of a basic T-gate and gate-head extended HEMT.

#### 3.2.1. Simulation of the DC Characteristics

We simulated and analyzed the DC characteristics of basic T-gate and gate-head extended HEMTs. Figure 5a,b show the $I_{DS}\text{–}V_{GS}$ transfer characteristics simulated at $V_{DS}$ values of 10 and 20 V, respectively. Additionally, $L_{Gate\text{-}Head\text{-}Top}$ and $L_{Gate\text{-}Head\text{-}Bottom}$ increased, and $I_{dss}$ and $G_{m}$ increased slightly. However, almost no significant changes are observed, and $V_{th}$ remains constant at −4.3 V, as shown in Figure 5a.

Figure 5b confirms that drain currents and $g_{m}$ values simulated at a $V_{DS}$ of 20 V were smaller than those obtained at a $V_{DS}$ of 10 V because the generated SHE was more at a higher $V_{DS}$. As shown in Figure 5c, the $I_{DS}\text{–}V_{DS}$ transfer characteristics were simulated at $V_{GS}$ values of −5, −4, −3, −2, −1, and 0 V. As the drain voltage increases, the drain current decreases owing to the increase in heat generation and electron scattering.

**Figure 6:**
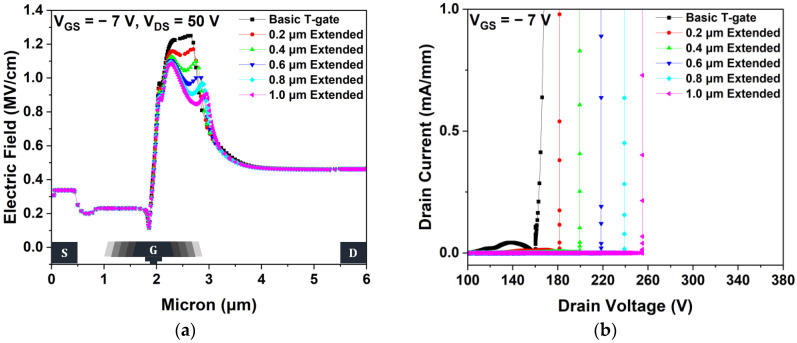
Simulations using different gate-head extended structures: (**a**) electric field distributions across the two-dimensional electron gas channel layer between the source and drain electrodes at V_GS_ = −7 V and V_DS_ = 50 V and (**b**) breakdown voltage characteristics at V_GS_ = −7 V.

Figure 6a shows the electric field distributions in the 2-DEG channel layer of the basic T-gate and gate-head extended HEMTs. The electric fields of the gate-head extended HEMT were redistributed, thereby reducing the peak electric field. Generally, impact ionization can be avoided by reducing the concentrated electric fields at the drain-side gate edge. Additionally, improving the $V_{BD}$ characteristics can be a possible solution for preventing severe impact ionization [34]. Furthermore, $V_{BD}$ was simulated at a gate voltage of −7 V to completely turn off the channel, and $V_{BD}$ was determined under a drain leakage current of 1 mA/mm. As shown in Figure 6b, the basic T-gate HEMT has a $V_{BD}$ of 167.78 V, and the 0.2, 0.4, 0.6, 0.8, and 1.0 μm extended HEMTs have $V_{BD}$ values of 181.50, 199.45, 218.46, 239.03, and 255.11 V, respectively. The maximum $V_{BD}$, which is 52.05% higher than that of a basic T-gate HEMT, was obtained from the 1.0 μm extended HEMT.

#### 3.2.2. Simulation of RF Characteristics

**Figure 7:**
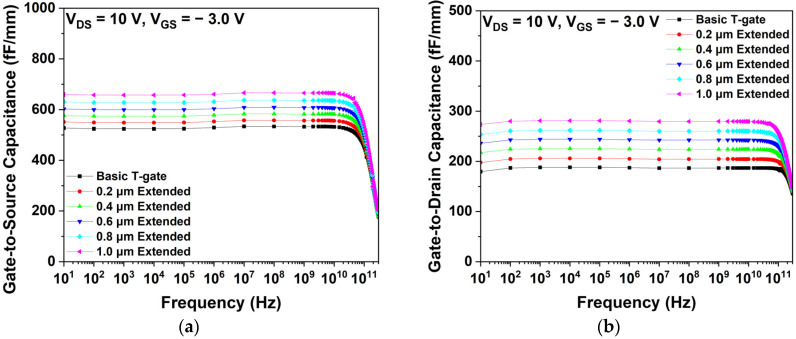
Capacitance characteristics as a function of frequency for different gate extended structures: (**a**) gate-to-source capacitances and (**b**) gate-to-drain capacitances.

As shown in Figure 7, $C_{gs}$ and $C_{gd}$ of the basic T-gate and gate-head extended HEMTs at a $V_{DS}$ of 10 V and a $V_{GS}$ of −3 V are compared. The increase in $L_{Gate\text{-}Head\text{-}Top}$ and $L_{Gate\text{-}Head\text{-}Bottom}$ resulted in increases in $C_{gs}$ and $C_{gd}$.

The relationship between capacitance and distance is expressed in Equation (6). Additionally, $C_{gs}$ is confirmed to typically be higher than $C_{gd}$ because $L_{gs}$ is shorter than $L_{gd}$, as shown in Table 1. In Figure 7a,b, we observe that the highest $C_{gs}$ and $C_{gd}$ are obtained in the 1.0 μm extended structure, because $L_{gs}$ and $L_{gd}$ were reduced as the gate-head extended structure was applied.

**Figure 8:**
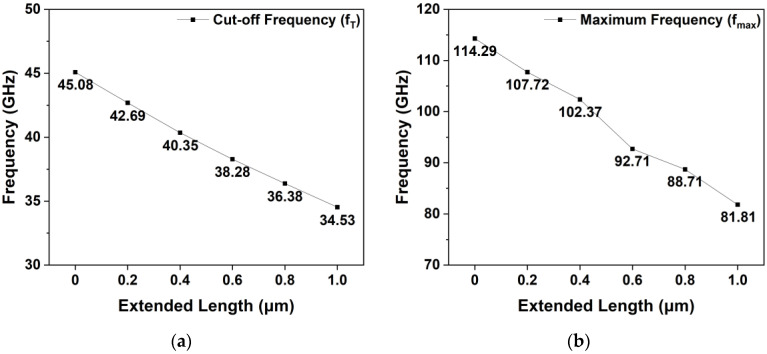
Simulated frequency values of the gate-head extended HEMTs at $V_{DS}$ = 10 V and $V_{GS}$ = −3 V: (**a**) $f_{T}$ and (**b**) $f_{max}$.

Figure 8 depicts the simulated $f_{T}$ and $f_{max}$ obtained using the basic T-gate and gate-head extended HEMTs, which were simulated at a $V_{DS}$ of 10 V and a $V_{GS}$ of −3 V. The simulated $f_{T}$ of the basic T-gate HEMT was 45.08 GHz, and those of the 0.2, 0.4, 0.6, 0.8, and 1.0 μm extended HEMTs were 42.69, 40.35, 38.28, 36.38, and 34.53 GHz, respectively. Furthermore, $f_{T}$ tended to decrease as $L_{Gate\text{-}Head\text{-}Top}$ and $L_{Gate\text{-}Head\text{-}Bottom}$ increased with the increase in $C_{gs}$.

The $f_{max}$ value of the basic T-gate HEMT was 114.29 GHz, and those of the 0.2, 0.4, 0.6, 0.8, and 1.0 μm extended HEMTs were 107.72, 102.37, 92.71, 88.71, and 81.81 GHz, respectively. According to Equation (5), the $f_{max}$ of the gate-head extended HEMTs decreased because $f_{max}$ is a function of $C_{gd}$ and $f_{T}$. Consequently, we observed that $f_{T}$ and $f_{max}$ considerably deteriorated by 30.55% and 39.70%, respectively, as the gate-extended HEMTs were applied.

### 3.3. Employment of a Gate-Recessed Structure in the 1.0 μm Extended HEMT

We confirmed that the 1.0 μm extended HEMT exhibits the highest $V_{BD}$. However, it had the lowest $f_{T}$ and $f_{max}$ among the gate-head extended HEMTs.

**Figure 9:**
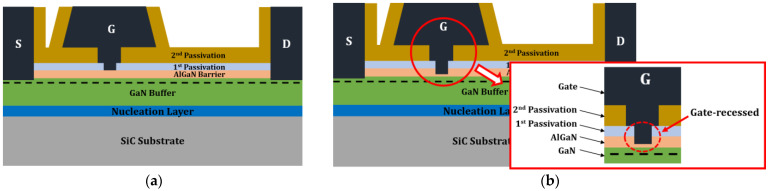
Cross-section schematics of the 1.0 μm extended HEMT: (**a**) non-gate-recessed structure and (**b**) gate-recessed structure.

We applied a gate-recessed structure to the 1.0 μm extended HEMT to further improve $V_{BD}$ and prevent the deterioration of frequency characteristics such as $f_{T}$ and $f_{max}$. Figure 9 shows the schematic of the 1.0 μm extended HEMT and 1.0 μm extended HEMT with the gate-recessed structure. The thickness of the 25 nm-thick AlGaN barrier layer was reduced to 13 nm in 3 nm steps to form the gate-recessed structure. Except for the gate-recessed depth, all the remaining geometrical parameters of the device were fixed. Subsequently, we simulated and analyzed the operational characteristics of the gate-recessed structures to determine the optimum gate-recessed depth.

#### 3.3.1. Simulation of DC Characteristics

**Figure 10:**
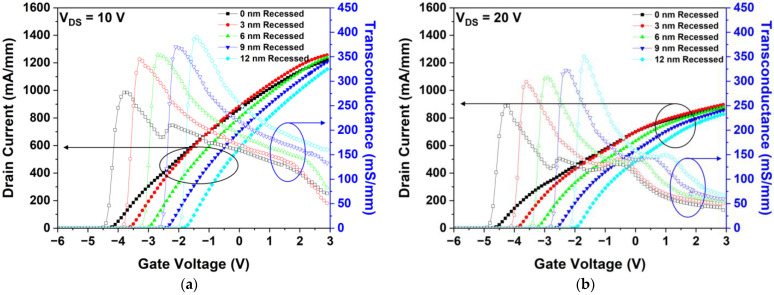
Simulated DC characteristics with gate-recessed depths between 0 to 12 nm: $I_{DS}\text{–}V_{GS}$ transfer characteristics at (**a**) $V_{DS}$ = 10 V; and (**b**) $V_{DS}$ = 20 V.

First, we simulated the DC characteristics, such as $I_{DS}\text{–}V_{GS}$ transfer characteristics, 2-DEG density, and $V_{BD}$, by increasing the gate-recessed depth. Figure 10a,b show the $I_{DS}\text{–}V_{GS}$ transfer characteristics simulated at $V_{DS}$ values of 10 and 20 V, respectively. The $G_{m}$ of the gate-recessed structure increased as the distance between the gate electrode and channel decreased [35]. According to Equation (7), $V_{th}$ shifted positively as the thickness of the AlGaN barrier under the gate electrode decreased [36].

(7) $$V_{th}-\emptyset_{eff}^{b}-∆E_{C}-\frac{qN_{S}{d_{AlGaN}}^{2}}{2\cdot \varepsilon_{AlGaN}}-\sigma \frac{d_{AlGaN}}{\varepsilon_{AlGaN}},$$

where $\emptyset_{eff}^{b}$ denotes the barrier height of the Schottky contact, $∆E_{C}$ is the discontinuity of the conduction band, q is the electron charge, $N_{S}$ is the 2-DEG concentration, and σ is the polarization charge density. The parameters associated with AlGaN are $d_{AlGaN}$ and $\varepsilon_{AlGaN}$, indicating the thickness and permittivity of the AlGaN, respectively.

As shown in Figure 10a, the $V_{th}$ of the 0, 3, 6, 9, and 12 nm recessed structures were −4.3, −3.7, −3.1, −2.5, and −1.9 V, respectively, and the simulated values of $G_{m}$ were 276.89, 344.91, 353.99, 370.06, and 389.84 mS/mm, respectively. The $G_{m}$ of the 12 nm recessed structure improved by 40.79% with respect to that of the non-gate-recessed structure. In Figure 10b, when a $V_{DS}$ of 20 V is applied, the severe SHE affects the reduction of the drain current. Additionally, $V_{th}$ and $G_{m}$ were confirmed to exhibit similar tendencies to those of the corresponding simulated parameters at a drain voltage of 10 V.

**Figure 11:**
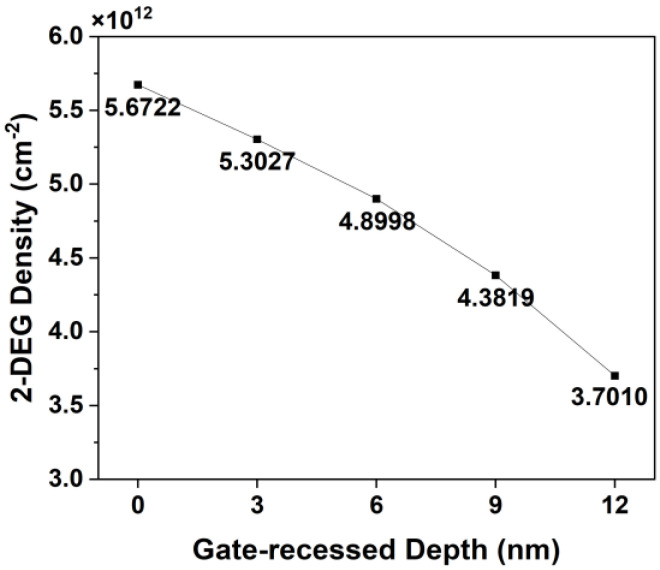
Simulated 2-DEG density as a function of gate-recessed depth.

Figure 11 shows the simulated 2-DEG densities of the five different gate-recessed structures simulated under the zero-bias condition. The 2-DEG density was observed to decrease as the gate-recessed depth increased; the 2-DEG density as a function of AlGaN barrier thickness was consistent with the theoretical calculation reported in previous studies [37,38,39].

**Figure 12:**
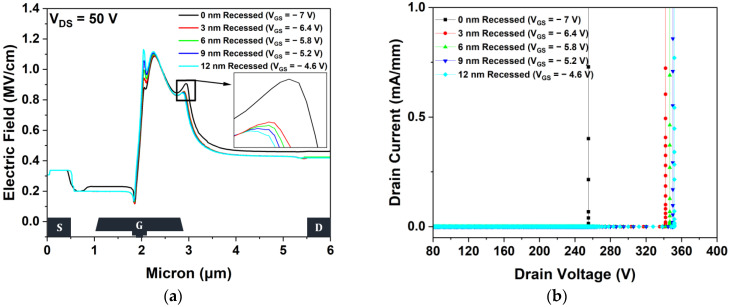
(**a**) Electric field distributions in the 2-DEG channel layer between the source and drain electrodes at $V_{DS}$ = 50 V under the pinch-off condition. The inset figure displays electric filed distributions between 2.825 to 3.025 μm and (**b**) off-state breakdown characteristics.

Figure 12a shows the electric field distributions in the 2-DEG channel layer of the 0, 3, 6, 9, and 12 nm recessed structures. Considering the $V_{th}$ shift, the electric fields of the structures were simulated at $V_{GS}$ values of −7, −6.4, −5.8, −5.2, and −4.6 V. The gate-recessed structure can redistribute the electric fields that are concentrated at a drain-side gate edge, unlike the non-gate-recessed structure. However, when the results obtained from the 3, 6, 9, and 12 nm recessed structures were compared, no significant changes were found in the electric field distributions. Figure 12b shows the $V_{BD}$ values of the five different gate-recessed structures simulated at a pinch-off. The $V_{BD}$ values of the 0, 3, 6, 9, and 12 nm recessed structures were 255.11, 342.03, 346.83, 350.33, and 351.85 V, respectively. The decrease in the electric field increased $V_{BD}$. Additionally, the recessed gate electrode in the AlGaN barrier results in close proximity between the gate and the channel, which improves carrier density. This increased carrier density generates a large depletion region around the gate and can increase the breakdown voltage due to the existence of a high potential barrier that helps prevent impact ionization. The largest $V_{BD}$ was observed in a 12 nm recessed structure, which was improved by 37.92% with respect to that of the non-gate-recessed structure.

#### 3.3.2. Simulation of the RF Characteristics

**Figure 13:**
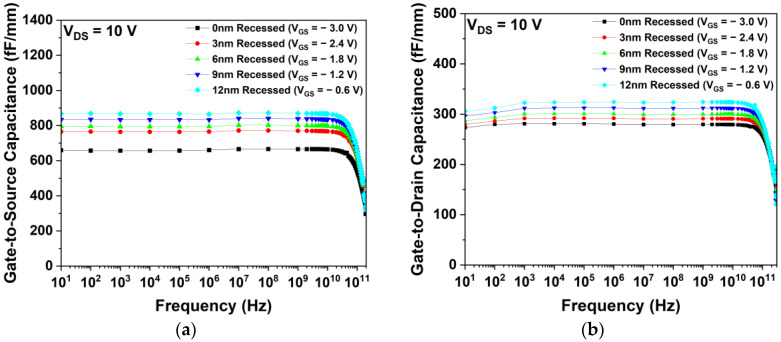
Capacitance characteristics as a function of frequency for different gate-recessed structures: (**a**) gate-to-source capacitances and (**b**) gate-to-drain capacitances.

The RF characteristics were simulated to identify the optimized gate-recessed depth, aiming to improve the frequency characteristics, such as $f_{T}$ and $f_{max}$. Additionally, $C_{gs}$ and $C_{gd}$ were simulated at a $V_{DS}$ of 10 V. Considering the positive shift of $V_{th}$, different $V_{GS}$ values of −3.0, −2.4, −1.8, −1.2, and −0.6 V were applied when simulating the 0, 3, 6, 9, and 12 nm recessed structures, respectively. As shown in Figure 13, the gate-recessed depth, $C_{gs}$, and $C_{gd}$ increased.

**Figure 14:**
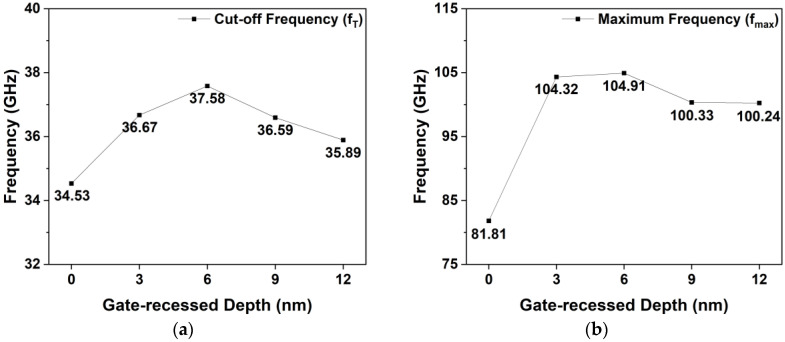
Simulated values of non-gate-recessed and gate-recessed structures: (**a**) $f_{T}$; (**b**) $f_{max}$.

Furthermore, $f_{T}$ and $f_{max}$ are simulated as shown in Figure 14, and the same $V_{GS}$ and $V_{DS}$ as those mentioned above are applied. Both $f_{T}$ and $f_{max}$ of the gate-recessed structures were higher than that of the non-gate-recessed structures. Furthermore, as the recessed depth increased, $f_{T}$ and $f_{max}$ increased up to a certain depth. However, beyond that depth, both $f_{T}$ and $f_{max}$ started to decrease. The largest $f_{T}$ and $f_{max}$ values of 37.58 and 104.91 GHz were obtained in the 6 nm recessed structure, and these values were improved by 8.83% and 28.24%, respectively, with respect to those of the non-gate-recessed structure. These results were directly affected by the increased value of $g_{m}$ owing to the decrease in the distance between the gate electrode and the channel. Ideally, the increase in $C_{gs}$, $C_{gd}$, and $g_{m}$ compensated for each other, resulting in constant values of $f_{T}$; increases in $C_{gs}$ and $C_{gd}$ of the active gate region decrease the influence of parasitic capacitance components. Therefore, $g_{m}$ has a dominant effect in determining the value of $f_{T}$. However, at a certain point, when the rate of increase of $g_{m}$ reduces progressively, $g_{m}$ is no longer a major factor in determining $f_{T}$ and $f_{max}$. Thus, frequency characteristics such as $f_{T}$ and $f_{max}$ can be improved by applying a gate-recessed structure [13,40]. Consequently, we suggest that the 6 nm recessed structure is the optimized one, considering high $V_{BD}$ and remarkable $f_{T}$ and $f_{max}$.

## 4. Discussion

In this study, we simulated and analyzed the DC and RF characteristics of 0.18 μm T-gate AlGaN/GaN HEMTs by symmetrically increasing $L_{Gate\text{-}Head\text{-}Top}$ and $L_{Gate\text{-}Head\text{-}Bottom}$. We confirmed the trade-off relationship between $V_{BD}$ and frequency characteristics. The 1.0 μm gate-head extended HEMT exhibited a remarkable $V_{BD}$, but the frequency characteristics, such as $f_{T}$ and $f_{max}$, were severely deteriorated. Unlike that of the basic T-gate structure, the $V_{BD}$ of the gate-head extended structure increased by 52.05%, whereas $f_{T}$ and $f_{max}$ decreased by 30.55% and 39.70%, respectively.

Therefore, the gate-recessed structure was applied to the 1.0 μm extended HEMT to minimize the degradation of frequency characteristics. The gate-recessed depth of the 6 nm structure demonstrated the lowest $f_{T}$ and $f_{max}$ degradation, with further improvement in $V_{BD}$. Consequently, $V_{BD}$ improved by 106.72% with respect to that of the basic T-gate HEMT, whereas $f_{T}$ and $f_{max}$ were only reduced by 19.96% and 8.94%, respectively. The proposed AlGaN/GaN HEMT structure shows a notable enhancement in $V_{BD}$ [41].

## 5. Conclusions

This paper presents a simulation study on 0.18 μm T-gate AlGaN/GaN HEMTs, in which the gate-head lengths are increased, and a gate-recessed structure is employed. To propose the optimized gate-head structure, I–V transfer curves, capacitances, and frequency characteristics were simulated. Before simulating the various HEMT structures, all simulation parameters were precisely set by matching with the measured data obtained from the fabricated HEMT to ascertain the reliability of the simulated results. Finally, we propose a 1.0 μm extended HEMT with a 6 nm gate-recessed structure as the optimized device by considering the trade-off relationship between $V_{BD}$ and the frequency characteristics. The optimized device demonstrated a significant improvement in $V_{BD}$ and an acceptable degradation of frequency characteristics with respect to the basic T-gate structure. Consequently, the simulation results prove that the proposed structure is a promising candidate for high-power applications.

## Figures and Tables

**Table 1:** Geometrical parameters of the 0.18 μm T-gate AlGaN/GaN HEMT.

Parameter	Value (μm)
① $L_{Source\text{-}Drain}$	5
② $L_{Gate\text{-}Source}$	1.05
③ $L_{Gate\text{-}Drain}$	3.15
④ $L_{Gate\text{-}Foot}$	0.18
⑤ $L_{Gate\text{-}Middle}$	0.34
⑥ $L_{Gate\text{-}Head\text{-}Bottom}$	0.8
⑦ $L_{Gate\text{-}Head\text{-}Top}$	0.6
⑧ $L_{Sidewall}$	0.2
Nucleation layer	0.2
GaN buffer	2
AlGaN barrier	0.025
1st passivation	0.05
2nd passivation	0.25

**Table 2:** Material parameters at room temperature applied in the simulation.

Parameters	Units	GaN	AlGaN
Bandgap energy	eV	3.39	3.88
Electron affinity	eV	4.2	2.3
Relative permittivity	-	9.5	9.38
Low field electron mobility	cm^2^/V-s	1500	300
High field electron mobility	-	GANSAT mobility model	
Saturation velocity	cm/s	$1.91\times 10^{7}$	$1.12\times 10^{7}$
Shockley-Read-Hall lifetime	s	$5.67\times 10^{12}$	$5.67\times 10^{12}$
Thermal conductivity constant	W/cm-K	1.3	0.4
Thermal conductivity factor	-	0.43	0
Acceptor trap doping	/${cm}^{3}$	$10^{18}$	-

## Data Availability

Data are contained within the article.
